# The Effect of Tauroursodeoxycholic Acid (TUDCA) Treatment on Pregnancy Outcomes and Vascular Function in a Rat Model of Advanced Maternal Age

**DOI:** 10.3390/antiox11071275

**Published:** 2022-06-28

**Authors:** Mazhar Pasha, Raven Kirschenman, Amy Wooldridge, Floor Spaans, Christy-Lynn M. Cooke, Sandra T. Davidge

**Affiliations:** 1 Department of Physiology, University of Alberta, Edmonton, AB T6G 2R3, Canada; pasha1@ualberta.ca; 2 Department of Obstetrics and Gynecology, University of Alberta, Edmonton, AB T6G 2S2, Canada; raven@ualberta.ca (R.K.); alwooldr@ualberta.ca (A.W.); floortje@ualberta.ca (F.S.); christyl@ualberta.ca (C.-L.M.C.); 3 Women and Children’s Health Research Institute, University of Alberta, Edmonton, AB T6G 1C9, Canada

**Keywords:** advanced maternal age, pregnancy outcomes, endoplasmic reticulum stress, tauroursodeoxycholic acid, wire myography, endothelium-dependent relaxation

## Abstract

Advanced maternal age (≥35 years) increases the risk of vascular complications in pregnancy that can result in fetal growth restriction and preeclampsia. Endoplasmic reticulum (ER) stress has been linked to adverse pregnancy outcomes in these complicated pregnancies. However, the role of ER stress in advanced maternal age is not known. We hypothesize that increased ER stress contributes to altered vascular function and poor pregnancy outcomes, and that treatment with the ER-stress inhibitor TUDCA will improve pregnancy outcomes. First, young and aged non-pregnant/pregnant rats were used to assess ER stress markers in mesenteric arteries; mesenteric artery phospho-eIF2α and CHOP expression were increased in aged dams compared to young dams. In a second study, young and aged control and TUDCA-treated dams were studied on gestational day (GD) 20 (term = 22 days). TUDCA treatment was provided via the drinking water throughout pregnancy (GD0-GD20; calculated dose of 150 mg/kg/day TUDCA). ER stress markers were quantified in mesenteric arteries, blood pressure was measured, pregnancy outcomes were recorded, mesenteric and main uterine arteries were isolated and vascular function was assessed by wire myography. Aged dams had increased phospho-eIF2α and CHOP expression, reduced fetal weight, reduced litter size, and impaired uterine artery relaxation. In the aged dams, TUDCA treatment reduced phospho-eIF2α and CHOP expression, reduced blood pressure, improved fetal body weight, and tended to improve uterine artery function compared to control-treated aged dams. In conclusion, our data illustrate the role of ER stress, as well as TUDCA as a potential therapeutic that may benefit pregnancy outcomes in advanced maternal age.

## 1. Introduction

Advanced maternal age is defined as maternal age ≥ 35 years at the time of delivery. Pregnancy at an advanced age is becoming increasingly more common in developed countries, accounting for 14–18% of total live births in North America [1,2,3]. Several studies have shown that advanced maternal age increases the risk of pregnancy complications, such as fetal growth restriction, preeclampsia, hypertension, gestational diabetes, preterm birth, small for gestational age infants, and stillbirth [4,5,6]. It is known that maternal vascular adaptations to pregnancy play an essential role in normal fetal growth and development [7,8,9,10]. In a rat model of advanced maternal age, we have demonstrated adverse pregnancy outcomes and a reduced capacity to sustain a pregnancy, reduced fetal weight, altered vascular function in systemic arteries, and increased active myogenic responses in both mesenteric and uterine arteries from aged dams compared to young dams [11,12]. In addition, other animal models of advanced maternal age (mouse and vervet/African green monkey) have also shown impaired vascular adaptations during pregnancy with advanced maternal age, that were associated with poor pregnancy outcomes and altered vascular function [13,14]. Although pregnancies at advanced maternal age are considered high-risk clinically, the underlying pathophysiological molecular mechanisms associated with the adverse pregnancy outcomes, particularly related to vascular dysfunction in advanced maternal age are not well established.

It is known that the process of aging is associated with impaired vascular endothelial function [15]. Among the various complex mechanisms, emerging studies show that an increase in endoplasmic reticulum (ER) stress and increased levels of reactive oxygen species (ROS) are associated with subsequent endothelial dysfunction in (non-pregnant) aging vessels [16,17]. The ER is a major site for the folding and trafficking of both intracellular and secretory proteins, and any disturbance in the protein-folding environment can lead to the accumulation of misfolded or unfolded proteins, triggering the evolutionarily conserved unfolded protein response (UPR). The main purpose of the UPR is to restore ER homeostasis for cell survival (adaptive/survival pathway) by increasing the synthesis of molecular chaperones involved in protein folding and/or degradation of misfolded proteins. The UPR is initiated via activation of one of the three signaling pathways/sensor proteins: protein kinase RNA-like endoplasmic reticulum kinase (PERK), inositol-requiring enzyme-1α (IRE1α), or activating transcription factor 6α (ATF6). These three sensors are held in an inactive state under normal conditions by binding glucose-regulated protein 78 (GRP78), a master regulator of ER stress. When ER stress occurs, GRP78 uncaps from the three sensors leading to the activation of UPR signaling pathways, initiating a downstream signaling cascade causing autophosphorylation of the sensor molecules PERK and IRE-1α. PERK activation leads to phosphorylation of eukaryotic translation-initiation factor 2α (eIF2α), eventually reducing global protein synthesis [18,19]. Similarly, IRE1α activation causes splicing of X-box binding protein-1 (XBP-1), a transcription factor that upregulates gene transcription for proteins involved in ER homeostasis [20,21]. ATF6 activation leads to its translocation to the Golgi apparatus, where it is cleaved and translocates to the nucleus to regulate the transcription of C/EBP homologous protein (CHOP) and XBP-1 [22]. However, if the adaptive/survival pathway fails, then prolonged ER stress leads to the accumulation of unfolded proteins, which may activate signaling pathways leading to apoptosis (PERK via CHOP or IRE-1α via c-Jun N-terminal kinase pathways). Thus, any cellular stress that interrupts protein folding can be a threat to cell viability [16].

Several studies have reported ER stress to play a role in adverse outcomes during pregnancy. For example, increased ER stress negatively affects blastocyst formation and decreases blastocyst development [23], and reduced mammalian and porcine oocyte maturation and embryo development in an in vitro culture system [24]. Further, increased placental ER stress has been associated with fetal growth restriction and early-onset preeclampsia [25,26]. In addition, ER stress is tightly linked to oxidative stress. Protein folding in the ER requires a tightly controlled redox environment and excess ROS generation can severely impact cellular homeostasis [22,26]. There are several intracellular processes that lead to ROS generation, but one of the key enzymes involved in ROS production in aging dams is NADPH oxidases (NOX) [27,28]. NOX was also shown to be the primary source of ROS associated with vascular dysfunction in resistance arterioles [29]. Accumulating research showed that ER stress increases ROS production via the NOX family (especially the NOX-2 and NOX-4 isoforms) [30,31,32]. Further, NOX-induced oxidative stress is also recognized as a key factor in the pathogenesis of adverse pregnancy outcomes such as fetal growth restriction and preeclampsia [33,34]. Overall, these studies suggest that ER and oxidative stress can have a negative effect on pregnancy. However, whether increased ER stress also contributes to impaired pregnancy outcomes and altered vascular adaptations in pregnancies at advanced maternal age is not known.

In the current study, we first assessed if maternal aging was associated with increased vascular ER stress, followed by a second set of experiments to evaluate the potential benefit of an ER stress inhibitor; tauroursodeoxycholic acid (TUDCA) on vascular function and pregnancy outcomes in a rat model of advanced maternal age. TUDCA is a bile acid derivative that occurs naturally in the body and is used to treat cholelithiasis and cholestatic liver disease [35,36]. Both in vitro and in vivo studies have shown a beneficial effect of TUDCA on embryo survival [37,38]. TUDCA treatment improved the rate of two-cell embryo development to blastocysts by attenuating the expression of active XBP-1 [39]. Further, mouse embryos cultured in the presence of TUDCA and transferred to pseudo- pregnant foster mothers had improved implantation rates and the number of live pups per surrogate mouse compared to the control group [40]. However, whether treatment with TUDCA can improve pregnancy outcomes in advanced maternal age has not been investigated. Therefore, we hypothesized that (1) impaired vascular function is due to increased ER stress in advanced maternal age and (2) TUDCA treatment will improve vascular function and pregnancy outcomes in a rat model of advanced maternal age by reducing ER stress.

## 2. Materials and Methods

### 2.1. Animal Model and Experimental Design

Female and male (for breeding) Sprague Dawley rats (*n* = 8–10 /group) were purchased from Charles River (St Constant, QC, Canada) at 3 months of age, allowed to acclimatize for one week, and were housed in an animal care facility in a 14:10 h light: dark cycle and at an ambient temperature of 22 ± 1 °C. We used an established rat model of advanced maternal age [13], which utilizes young (3 to 4 months of age) and aged rats (9 to 10 months of age, corresponding to ~35 years of age in humans; considering milestones such as reproductive senescence, weaning, skeletal, and sexual maturity). Young rats were maintained on ad libitum access to standard rat chow, while aging rats were maintained on a controlled diet until pregnancy, which consist of 6 pellets of standard chow/day based on National Research Council recommendations, to prevent age-related obesity as a confounding factor [41,42]. For the pregnant groups, young and aged female rats (3–5 months and 9–10 months) were mated overnight with young male rats (3–5 months), and pregnancy was confirmed by the presence of sperm in a vaginal smear, which was considered as gestational day (GD) 0, after which all dams were maintained on ad libitum standard diet [13].

Two different experimental designs were used. The first study assessed the expression level of ER stress markers and NOX (NOX-2 and NOX-4 isoforms) in systemic arteries of young and aged groups. The experimental groups consisted of young non-pregnant and pregnant dams and aged non-pregnant and pregnant rats. On GD20 (term = 21–22 days), pregnant rats and aged-matched non-pregnant rats were euthanized using isoflurane (4% in oxygen) followed by exsanguination via cardiac puncture. The mesenteric arteries were excised and placed in ice-cold HEPES-buffered Physiological Saline Solution (PSS; in mmol/L: 142 NaCl, 1.56 CaCl_2_, 4.7 KCl, 1.17 MgSO_4_, 10 HEPES, 1.18 KH_2_PO_4_, and 5.5 glucose, pH 7.4), following which the mesenteric arteries were isolated for Western blot analysis. In the second study, we assessed if the ER stress inhibitor, TUDCA, could improve pregnancy outcomes and vascular function in pregnancy. For this study, pregnant young and aged rats, either control or TUDCA treated rats, were used. TUDCA treatment was provided via the drinking water throughout pregnancy (GD0-GD20; to a calculated dose of ~150 mg/kg/day TUDCA, based on previous studies [43,44,45]). The dose was calculated based on the average weight of the rats and their average daily water consumption. The control groups received regular drinking water. All TUDCA-treated rats were closely monitored for any signs of adverse drug reactions during the study period. On GD20 (term = 21–22 days), blood pressure was measured (see below), after which the rats were anesthetized using isoflurane and euthanized by exsanguination via cardiac puncture. The number of pups and resorption sites were recorded. Fetal biometrics, including crown-rump length (CR), abdominal girth (AG), body weight, and placental weight were collected. The mesenteric arcade and main uterine arteries were immediately excised and placed in ice-cold HEPES-buffered PSS for ex vivo vascular function (both uterine and mesenteric arteries) and Western blot analysis (mesenteric arteries), as described below.

### 2.2. Blood Pressure Measurements

In the TUDCA treatment study, on GD20, blood pressure was assessed by tail-cuff plethysmography (CODA High Throughput System, Kent Scientific, Torrington, CT, USA). Before pregnancy, all rats were trained in restraint nose cone holders for one week for acclimatization. On the day of the experiments, rats were placed in the restraint holders, and the occlusion tail cuff and volume pressure recording sensor cuff were placed close to the base of the tail. After a 20 min. acclimatization period, at least 10 consecutive blood pressure measurements (mean arterial pressure, systolic, and diastolic pressure) were recorded and averaged for each rat [46].

### 2.3. Mesenteric and Uterine Artery Vascular Function by Wire Myography

In the TUDCA treatment study, vascular function was assessed ex vivo in mesenteric and uterine arteries using wire myography. The mesenteric and uterine vasculature were studied, for distinct reasons. Small resistance mesenteric arteries are important in regulating overall peripheral vascular resistance, whereas the uterine artery is known to undergo extensive remodeling during pregnancy and is critical in supporting normal growth and development. Second-order mesenteric and main uterine arteries were isolated in ice-cold PSS and mounted on an isometric myograph system (620 M Danish Myo Technology, Copenhagen, Denmark) using 40 µm tungsten wires. LabChart software (AD Instruments; Colorado Springs, CO, USA) was used to record the isometric tension of the arteries. Normalization of the arteries was performed via a series of stepwise increases in diameter to determine their optimal resting tension, set to 0.8 × IC100; 13.3 kPa for mesenteric arteries and 7.32 kPa for uterine arteries (the internal circumference equivalent to a transmural pressure of 100 mmHg). Following normalization, vascular integrity of the arteries was confirmed by exposing the vessels to a single dose of phenylephrine (10 µmol/L; Sigma-Aldrich, St Louis, MO, USA) for 5 min. Following a washout period with PSS, arteries were allowed to rest for 10 min, after which they were exposed to a second single dose of phenylephrine (10 µmol/L followed by a single dose of methylcholine (MCh; 3 µmol/L; Sigma-Aldrich). After this wakeup procedure, the vessels were allowed to rest for 30 min, and endothelium-dependent vascular responses to MCh were investigated using a cumulative dose-response curve to MCh (0.003 to 3 µmol/L) after pre-constriction with phenylephrine (3 µmol/L) to produce 80% of the maximal response. To assess the contribution of nitric oxide (NO) to vasodilation, part of the arteries was pre-incubated with the pan-NO synthase inhibitor N(G)-nitro-L-arginine methyl ester hydrochloride (L-NAME; 100 µmol/L; Sigma-Aldrich) 30 min prior to the start of the cumulative dose-response curve to MCh. Concurrently, in a separate vessel segment, endothelium-independent relaxation responses to sodium nitroprusside (SNP; a NO donor; 0.003 to 2 µmol/L: Sigma-Aldrich) were assessed. At the end of each experiment, all arteries were exposed to a 124 mmol/L potassium chloride solution (high KCl buffer; containing in mmol/L: 24 NaCl, 4.9 CaCl_2_, 124 KCl, 2.4 MgSO_4_, 10 HEPES, 1.18 KH_2_PO_4_, and 5.5 glucose; pH 7.4) to determine maximum vasoconstriction responses. All data was recorded using LabChart software (AD Instruments; Colorado Springs, CO, USA) and were summarized as maximum vasodilation responses to MCh/SNP (Emax), sensitivity to MCh/SNP, defined as the negative log of the mean effective concentration that produces 50% of the maximal response (pEC50), the area under the curve (AUC), and ΔAUC. GraphPad Prism 9 (GraphPad Software, San Diego, CA, USA) was used to analyze the data.

### 2.4. Expression of Oxidative and ER Stress Markers Using Western Blot Analysis

Snap frozen mesenteric arteries were homogenized using cell lysis buffer (concentration in mmol/L: 5 EDTA, 10 sodium pyrophosphates tetrabasic, 100 sodium, 9-fluoride with 1% Nonidet P-40, and 20 Tris, pH 7.4) containing phosphatase inhibitor (2 mmol/L sodium orthovanadate, Sigma), 1 mmol/L Phenylmethylsulfonyl fluoride (PMSF; Fluka Biochemika, St. Louis, MO, USA), and a protease inhibitor cocktail (Thermo Scientific, Waltham, MA, USA). A bicinchoninic acid assay (Pierce, Rockford, IL, USA) was used to determine the total protein concentration of the samples. Based on total protein concentration, tissue homogenates (50 μg of protein) were loaded and separated on 10% and 12% SDS-polyacrylamide gels and transferred to a nitrocellulose membrane (100 V, 2 h: 0.2 µm, Bio-Rad, Hercules, CA, USA). For normalization, total protein quantification was performed using LI-COR Revert 700 Total Protein Stain and imaged using the LI-COR Odyssey system. Followed by incubating the membranes with BlockOut^®^-Universal Blocking Buffer (Rockland, PA, USA) for 1 h. All membranes were incubated overnight at 4 °C with primary antibodies for NOX-2 (1:500 mouse monoclonal, Santa Cruz Biotechnology, Dallas, TX, USA), NOX-4 (1:500 rabbit polyclonal, Proteintech), GRP78 (1:1000 rabbit polyclonal, Cell Signaling Technology, Danvers, MA, USA), phospho-eIF2α (1:500 rabbit polyclonal, Cell Signaling Technology), Total-eIF2α (1:500 mouse monoclonal, Santa Cruz Biotechnology), CHOP (1:500 mouse monoclonal, Cell Signaling Technology), sXBP1 (1:500 rabbit polyclonal, Cell Signaling Technology), or ATF-6 (1:1000 mouse monoclonal, Santa Cruz Biotechnology) in phosphate-buffered saline with Tween-20 (PBST in mmol/L; 2.7 KCl, 137 NaCl, 1.8 KH_2_PO_4_, 10 Na_2_HPO_4_, and Tween^®^ 20: 0.1% (*w*/*v*); Thermo Scientific). The following day, membranes were incubated with their corresponding secondary antibodies: IRDye donkey anti-rabbit IgG (NOX-2, NOX-4, GRP78, phopsho-eIF2α, sXBP-1) and IRDye donkey anti-mouse IgM (for Total-eIF2α, CHOP, and ATF-6) at 1:10,000 dilution in PBST buffer. At the end, blots were washed several times and were visualized with an LI-COR Odyssey Bioimager (LI-COR Biosciences, Lincoln, NE, USA) and quantified using ImageStudioLite software (LI-COR Biosciences). All data were normalized to total protein (except phosph-eIF2α, which was normalized to total-eIF2α) and expressed as percent change compared to the respective control group (young non-pregnant or young pregnant rats).

### 2.5. Statistical Analyses

All data were plotted and analyzed using GraphPad Prism 9 (GraphPad Software, San Diego, CA, USA) or Stata (StataCorp LLC, College Station, TX, USA) and presented as mean ± SEM. Statistical differences were tested using a two-way ANOVA with either planned contrast analysis or Sidak’s post-hoc test for multiple comparisons; *p* < 0.05 was considered statistically significant.

## 3. Results

### 3.1. Increased Expression of ER Stress Markers and NOX-4 in Aged Non-Pregnant and Pregnant Rats

**Figure 1:**
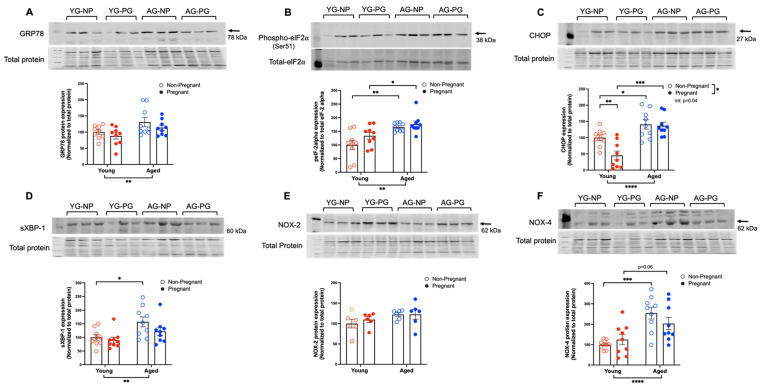
Increased expression of ER stress markers and NOX-4 in aged non-pregnant and pregnant rats. Expression levels of (**A**) GRP78, (**B**) phosph-eIF2α (Ser51), (**C**) CHOP, (**D**) sXBP-1, (**E**) NOX-2, and (**F**) NOX-4 proteins normalized to total protein in mesenteric arteries of young (3–4 months; in red) and aged (9–10 months; in blue) pregnant (gestational day 20; closed circles) and non-pregnant (age-matched; open circles) rats. Representative blots are shown above the graphs, and data are presented as mean ± SEM and expressed as a percentage of the control (i.e., the mean of the young non-pregnant group); analyzed by two-way ANOVA with Sidak’s multiple comparisons post-hoc test; *n* = 6–9/group. The number of asterisks defines the level of statistical significance observed among the data in the graphs: * *p* < 0.05, ** *p* < 0.01, *** *p* < 0.001, and **** *p* < 0.0001; main ANOVA effects are depicted below the x-axis (aging) or beside the legend (pregnancy); post-hoc test results are shown within the graphs. YG-NP = Young non-pregnant; YG-PG = Young pregnant; AG-NP = Aged non-pregnant; AG-PG = Aged pregnant.

In the first study, we assessed if the expression of ER stress markers and NOX in systemic (mesenteric) arteries was increased in advanced maternal age pregnancies. Both GRP78 and phosph-eIF2α expression were increased in the aged groups with no effect of pregnancy (Figure 1A,B). CHOP expression was increased in the aged groups, while the expression of CHOP was reduced in young pregnant rats compared to young non-pregnant rats, without differences between aged pregnant and non-pregnant rats (significant interaction; Figure 1C). sXBP-1 protein levels were higher only in the aged non-pregnant rats compared to the young non-pregnant group (Figure 1D). There were no changes in the expression of ATF-6 among the groups (data not shown). As ER stress increases ROS production via NOX, we measured the expression of two key isozymes NOX-2 and NOX-4 isoforms. There were no differences in NOX-2 protein expression between the groups (Figure 1E). However, NOX-4 protein expression was higher in aged non-pregnant rats compared to young non-pregnant rats and tended to be higher in aged dams compared to young dams (Figure 1F).

### 3.2. Reduced ER Stress Protein Expression in TUDCA-Treated Aged Dams

**Figure 2:**
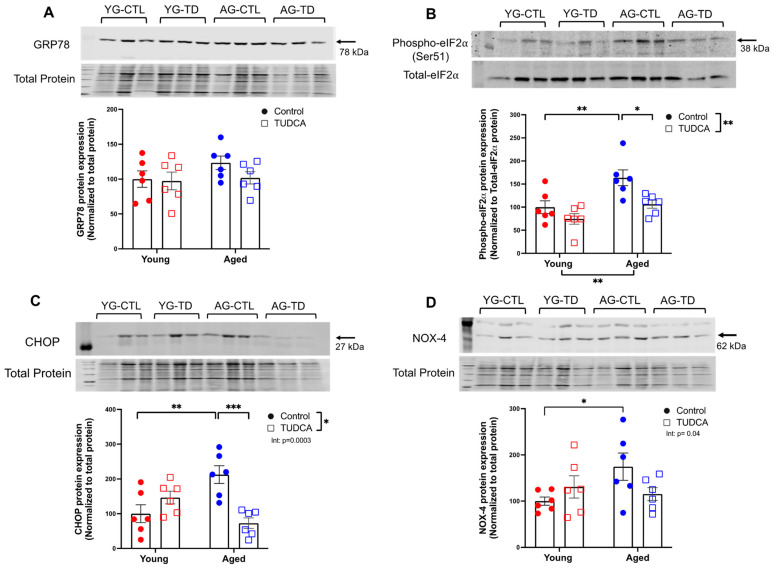
Reduced expression of ER stress markers in TUDCA-treated aged dams. Expression levels of (**A**) GRP78, (**B**) phosph-eIF2α (Ser51), (**C**) CHOP, and (**D**) NOX-4 proteins normalized to total protein in mesenteric arteries of young (3–4 months; in red) and aged dams (9–10 months; in blue) with (open squares) or without (closed circles) TUDCA-treatment on gestational day 20. Representative blots are shown above the graphs, and data are presented as mean ± SEM and expressed as a percentage of control (i.e., the mean of the young control dams); analyzed by two-way ANOVA with Sidak’s multiple comparisons post-hoc test; *n* = 6/group. The number of asterisks defines the level of statistical significance observed among the data in the graphs: * *p* < 0.05, ** *p* < 0.01, and *** *p* < 0.001; main ANOVA effects are depicted below the x-axis (aging) or beside the legend (TUDCA); post-hoc test results are shown within the graphs. YG-CTL = Young control dams; YG-TD = Young TUDCA-treated dams; AG-CTL = Aged control dams; AG-TD = Aged TUDCA-treated dams.

The second study was designed to assess if treatment with the ER stress inhibitor TUDCA could reduce the ER stress that had been shown to be increased in the systemic vasculature of the aged dams, as well as improve pregnancy outcomes and vascular function. No changes were seen in the expression of GRP78 protein between young and aged control and TUDCA-treated groups (Figure 2A). However, phospho-eIF2α and CHOP expression was increased in aged control dams compared to young control dams, and this was reduced by TUDCA treatment in the aged dams, without the effect of TUDCA treatment in the young dams (Figure 2B,C). NOX-4 expression was increased in the aged dams compared to young saline-treated dams, and this aging effect was no longer significant in the TUDCA-treated control compared to TUDCA-treated aged dams (significant interaction; Figure 2D).

### 3.3. Reduced Blood Pressure in Aged TUDCA-Treated Dams

**Figure 3:**
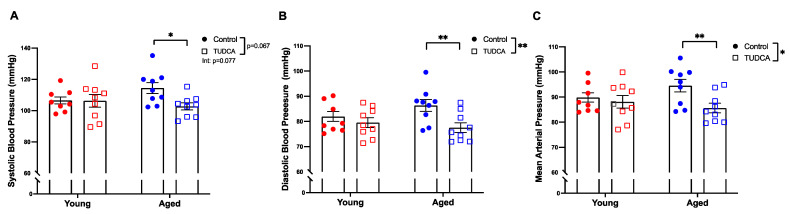
Blood pressure was reduced in TUDCA-treated aged dams. (**A**) Systolic, (**B**) diastolic, and (**C**) mean arterial blood pressure of young control (3–4 months; in red) and aged dams (9–10 months; in blue) with (open squares) or without (closed circles) TUDCA-treatment on gestational day 20. Data presented as mean ± SEM; analyzed by two-way ANOVA with Sidak’s multiple comparisons post-hoc test; *n* = 8–9/group. The number of asterisks defines the level of statistical significance observed among the data in the graphs: * *p* < 0.05 and ** *p* < 0.01; main ANOVA effects are depicted below the x-axis (aging) or beside the legend (TUDCA); post-hoc test results are shown within the graphs.

TUDCA treatment reduced blood pressure (systolic, diastolic, and mean arterial pressure) in only the aged dams (Figure 3A–C).

### 3.4. Mesenteric Artery Vascular Function Was Not Impacted by Age or TUDCA Treatment

**Figure 4:**
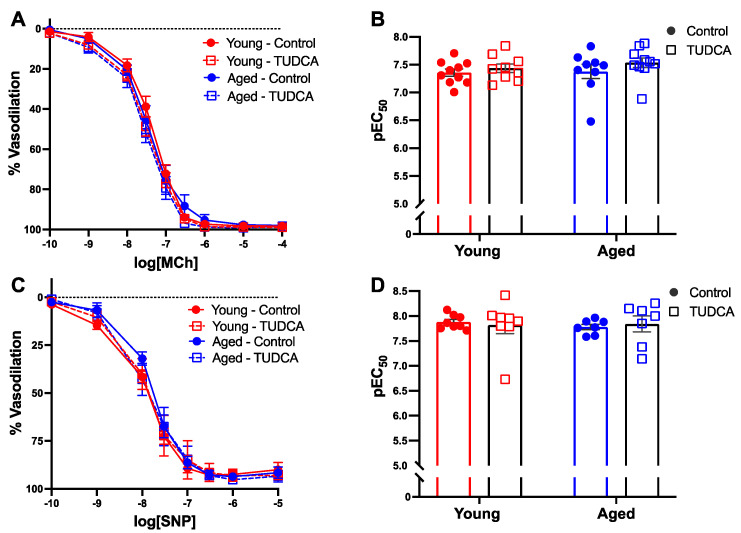
No differences in the endothelium-dependent or endothelium-independent vasodilation responses in mesenteric arteries between the groups. Endothelium-dependent vasodilation responses to increasing doses of (**A**) MCh or (**C**) SNP in mesenteric arteries of young control (3–4 months; in red) and aged dams (9–10 months; in blue) with (open squares) or without (closed circles) TUDCA-treatment on gestational day 20. (**B**,**D**) Data summaries of the sensitivity to MCh and SNP (pEC50). Data are presented as mean ± SEM; analyzed by two-way ANOVA with Sidak’s multiple comparisons post-hoc test; *n* = 8–9/group.

**Figure 5:**
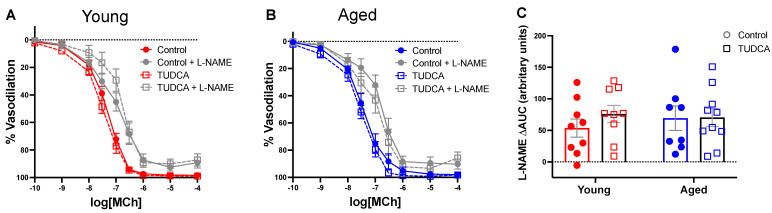
TUDCA did not change the contribution of nitric oxide to vasodilation in mesenteric arteries. (**A**,**B**) Endothelium-dependent vasodilation responses to increasing doses of MCh in the presence or absence of L-NAME in mesenteric arteries of young control (3–4 months; in red) and aged dams (9–10 months; in blue) with (open squares) or without (closed circles) TUDCA-treatment on gestational day 20. (**C**) Data summary of A + B, as differences in area under the curve (ΔAUC) in the presence or absence of L-NAME. Data are presented as mean ± SEM; analyzed by two-way ANOVA with Sidak’s multiple comparisons post-hoc test; *n* = 8–9/group.

In mesenteric arteries, no differences in MCh-induced endothelium-dependent vasodilation responses were observed between the groups (Figure 4A,B). In addition, there were no differences in endothelium-independent relaxation responses to SNP (Figure 4C,D). As NO is a potent vasodilator and plays an important role in regulating blood flow during pregnancy, we evaluated NO contribution using L-NAME and showed that NO contribution was similar in all the groups (Figure 5A–C).

### 3.5. Increased Fetal Body Weight in Aged TUDCA-Treated Dams

**Figure 6:**
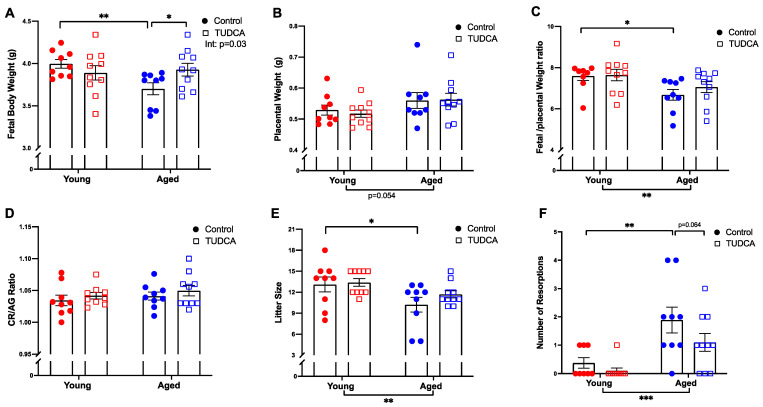
TUDCA treatment increased fetal body weight in aged dams. Pregnancy outcomes such as (**A**) fetal body weight, (**B**) placental weight, (**C**) fetal/placental weight ratio, (**D**) fetal crown-rump (CR)/abdominal girth (AG) ratio, (**E**) litter size, and (**F**) number of resorptions in young control (3–4 months; in red) and aged dams (9–10 months; in blue) with (open squares) or without (closed circles) TUDCA-treatment on gestational day 20. Data are presented as mean ± SEM; analyzed by two-way ANOVA with Sidak’s multiple comparisons post-hoc test; *n* = 9–10/group. The number of asterisks defines the level of statistical significance observed among the data in the graphs: * *p* < 0.05, ** *p* < 0.01, and *** *p* < 0.001; main ANOVA effects are depicted below the x-axis (aging) or beside the legend (TUDCA); post-hoc test results are shown within the graphs.

Fetal body weight was lower in control aged dams, while TUDCA treatment increased fetal body weight in the aged dams without any changes in the young control dams (Figure 6A). Placental weights were not different among the groups (Figure 6B). The fetal/placental weight ratio was decreased in control aged dams compared to young dams (Figure 6C). No differences in the crown-rump length/abdominal girth (CR/AG) ratio was observed between the groups (Figure 6D). Litter sizes were significantly reduced in control, but not TUDCA-treated aged compared to young dams (Figure 6E). The number of fetal resorptions was higher in the aged dams, which tended to be reduced with TUDCA treatment (Figure 6F).

### 3.6. TUDCA Treatment Tended to Improve Uterine Artery Function in Aged Dams

**Figure 7:**
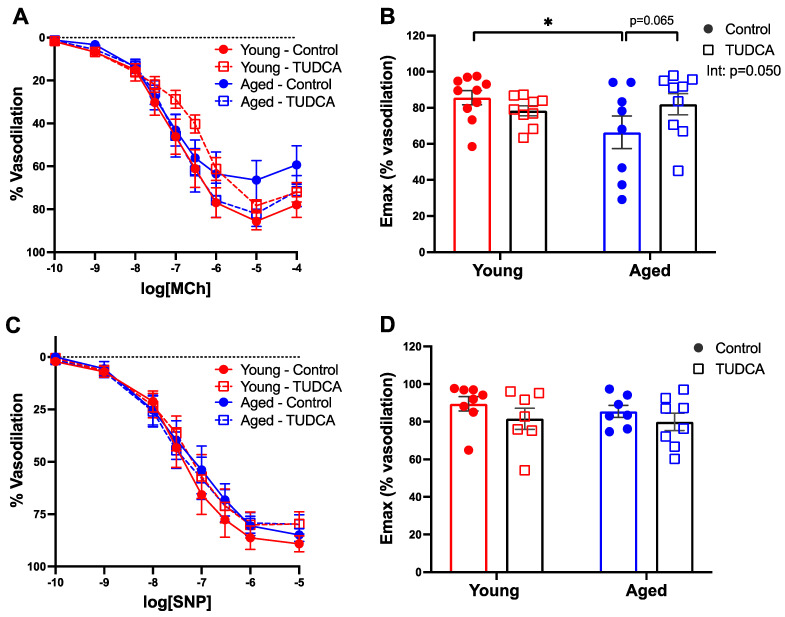
TUDCA tended to improve uterine artery endothelium-dependent vasodilation responses in aged dams. Endothelium-dependent and endothelium-independent vasodilation responses to increasing doses of (**A**) MCh or (**C**) SNP in uterine arteries of young control (3–4 months; in red) and aged dams (9–10 months; in blue) with (open squares) or without (closed circles) TUDCA-treatment on gestational day 20. (**B**,**D**) Data summaries of maximal vasodilation responses to MCh and SNP (Emax). Data are presented as mean ± SEM; analyzed by two-way ANOVA with Sidak’s multiple comparisons post-hoc test; *n* = 8–9/group. The number of asterisks defines the level of statistical significance observed among the data in the graphs: * *p* < 0.05; main ANOVA effects are depicted below the x-axis (aging) or beside the legend (TUDCA); post-hoc test results are shown within the graphs.

**Figure 8:**
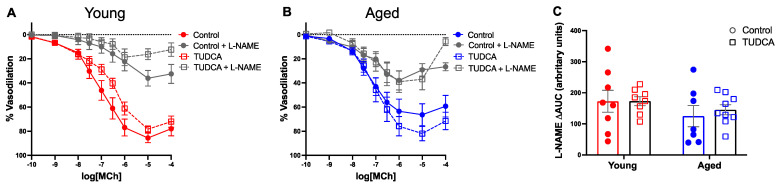
No effect of TUDCA treatment on nitric oxide contribution to vasodilation in uterine arteries between the groups. (**A**,**B**) Endothelium-dependent vasodilation responses to increasing doses of MCh in the presence or absence of the pan nitric oxide synthase inhibitor L-NAME in uterine arteries of young control (3–4 months; in red) and aged dams (9–10 months; in blue) with (open squares) or without (closed circles) TUDCA-treatment on gestational day 20. (**C**) Data summary of A + B, as differences in area under the curve (ΔAUC) in the presence or absence of L-NAME. Data are presented as mean ± SEM; analyzed by two-way ANOVA with Sidak’s multiple comparisons post-hoc test; *n* = 8–9/group.

MCh-induced maximum vasodilation responses were reduced in uterine arteries of aged control dams compared to young control dams, whereas TUDCA treatment tended to increase maximum vasodilation responses in aged dams, without effect in uterine arteries of the young dams (Figure 7A,B). There were no changes in uterine artery endothelium-independent vasodilation responses to SNP between the groups (Figure 7C,D). Moreover, pre-incubation with L-NAME revealed a similar NO contribution among the groups (Figure 8A–C).

## 4. Discussion

The main objective of the current study was to evaluate whether ER stress contributes to impaired vascular adaptations and pregnancy outcomes in advanced maternal age, and to assess whether targeting ER stress using TUDCA could reduce ER stress and improve vascular function and pregnancy outcomes. Our data showed that TUDCA treatment in a rat model of advanced maternal age reduced ER stress in mesenteric arteries, decreased blood pressure, increased fetal body weight, and tended to improve vasodilation responses in uterine arteries, suggesting a beneficial effect.

In a previous study, we observed altered vascular to pregnancy in mesenteric arteries of aged dams compared to young dams [14]. Given the association of ER stress with adverse pregnancy outcomes ([27,47], we speculated that ER stress may be involved in mediating this vascular dysfunction. In the current study, we assessed the expression of various ER stress markers and observed a higher expression of GRP78 in the aged rats compared to the young rats (main effect), but no changes in aged non-pregnant rats compared to young non-pregnant rats. Further, we observed an increased expression of phospho-eIF2α, CHOP, and sXBP-1 from aged non-pregnant rats compared to young non-pregnant rats. In general, under conditions of ER stress, GRP78 is released from the UPR sensors activating the downstream signaling pathways to regain ER homeostasis. One way of achieving homeostasis is by increasing the expression of phospho-eIF2α (to reduce the global protein synthesis) via the PERK pathway, however, prolonged ER stress leads to activation of pro-apoptotic factors such as CHOP. In the current study, a possible explanation for the differential expression of ER stress proteins (i.e., no changes in GRP78, but increased phospho-eIF2α and CHOP) could be that there was an increased expression of GRP78 at an earlier stage of the pregnancy, which eventually returned to basal conditions once it activated the downstream targets (the UPR response, phospho-eIF2α, and CHOP). This has been previously reported, for example, Kumar et al. observed differential regulation of ER stress markers (no changes in the expression of GRP78 together with increased CHOP expression) in anterior ischemic optic neuropathy in adult mice (1). Similarly, Karaskov et al. observed no changes in the levels of GRP78 but increased expression of phospho-eIF2α, CHOP, ATF4, and XBP1protein in INS-1 pancreatic β-cells exposed to palmitate (a known ER stress inducer) (2). An increased expression of ER stress proteins in aged non-pregnant rats is in line with aging literature [48,49,50,51] (i.e., an age-related increase in vascular ER stress). In addition, elevated levels of phospho-eIF2α and CHOP in aged dams compared to young dams suggest that the age-related increase in ER stress persists in pregnancy and could contribute to vascular dysfunction and impair pregnancy outcomes. Interestingly, CHOP expression was reduced in young pregnant dams compared to their non-pregnant controls, which was not seen in the aged rats, suggesting this may be a pregnancy adaptation that did not occur with advanced maternal age.

Evidence in the literature supports a strong interplay between ER and ROS, their association with vascular changes in aging vasculature [17,49,50], and NOX as the primary source of ROS [17,30,49,50]. Galan et al. showed an increased expression of ER stress proteins (phospho-eIF2α, CHOP, and ATF6), and NOX-2 and NOX-4 were associated with vascular dysfunction in mesenteric arteries and aortas using C57BL/6J (control) and p47phox^−/−^ mice (NOX lacking) injected with tunicamycin (an ER stress inducer) [51]. In addition, Lee et al. using C57BL/6J mice, and the NOX-4 KO mice model showed an increased expression of IRE1α and NOX-4 could be linked to vascular dysfunction in aging [52]. Thus, the increased expression of NOX-4 in the aged groups suggests upregulation of NOX-4 under ER stress conditions that could contribute to altered vascular function.

After finding signs of systemic vascular ER stress, together with (previously reported) impaired pregnancy outcomes and altered vascular function [14]; we wanted to assess if pregnancy outcomes and vascular function could be improved by ameliorating ER stress. To the best of our knowledge, we are the first to assess the effect of an ER stress inhibitor, such as TUDCA, on pregnancy outcomes and vascular function in a rat model of complicated pregnancy. TUDCA is a naturally occurring bile acid, and chemically, TUDCA is a taurine conjugate of ursodeoxycholic acid (UDCA), which is approved by the Food and Drug Administration for the treatment of primary biliary cholangitis and is also safely used in pregnancy to treat intrahepatic cholestasis [53,54,55]. Compared to UDCA, TUDCA is better absorbed by the intestine and liver because of its higher water solubility at various pHs [38,55]. In addition to its anti-cholestatic properties, it has been shown that TUDCA is an effective inhibitor of ER stress [56,57]. For instance, TUDCA improved endothelial dysfunction in both animal (hypertension and diabetic mouse models) [56,57] and clinical studies (Type 2 diabetes mellitus) [58]. Here, we showed that TUDCA treatment in aged dams reduced the expression of phospho-eIF2α, CHOP, and NOX-4 in systemic arteries. This is in line with other studies in both rats and mice which showed that endothelial dysfunction is associated with upregulation of pelF2α/ATF4/CHOP in mesenteric resistance arteries and aortas, albeit these studies were not performed during pregnancy [44,59,60]. Moreover, others have previously shown that TUDCA treatment improved vascular function by reducing ER stress and NOX-2 and NOX-4 expression in mesenteric arteries and aortas in a hypertension and diabetic mouse model (non-pregnant mice) [31,44,51]. Collectively, our data suggest that ER stress may contribute to impaired vascular adaptations in aged dams and that TUDCA treatment may have beneficial effects on ER/oxidative stress in the systemic vasculature.

Mesenteric resistance arteries play a significant role in the systemic circulation, regulating overall peripheral vascular resistance during normal pregnancy [61,62]. However, our data showed no differences in mesenteric artery endothelium-dependent vasodilation responses or NO contribution between the groups. These findings contrast with other studies, which showed that TUDCA increased NO bioavailability by reducing ER stress and improved vascular function in mesenteric arteries and aorta from tunicamycin-treated mice and in db/db mice [44,56]. A potential explanation for these differences in vascular outcomes after TUDCA treatment could be that the other studies were conducted in non-pregnant rodents, and chemically induced ER stress causes pronounced vascular endothelial dysfunction with reduced NO, which is not what we observed with advanced maternal age rats [14]. Further, the rat age of our model may not be sufficiently old enough to affect endothelium-dependent relaxation in mesenteric arteries, as it is possible that age-related vascular changes are ongoing. The latter was more evident in uterine arteries, with reduced uterine artery maximum vasodilation response and fetal growth restriction in the offspring (discussed below), implying that the vascular changes could be at a subthreshold level.

TUDCA reduced blood pressure in aged dams compared to untreated aged dams, such that blood pressure was similar to that of young dams. Although the effects of TUDCA on blood pressure in pregnancy are not well established, research in non-pregnant animal models has demonstrated that TUDCA, by alleviating ER stress (i.e., reduced expression of markers such as GRP78, phospho-eIF2α, CHOP, IRE1, XBP1, and ATF6) decreases blood pressure, reduces arterial stiffness, and improves endothelial dysfunction in spontaneously hypertensive rats [56,63,64]. It may be speculated that the reduction in ER stress that we observed contributed to the decrease in blood pressure by TUDCA in aged dams.

We hypothesized that TUDCA may be able to improve pregnancy outcomes in aged dams. Indeed, TUDCA treatment increased fetal body weight and tended to reduce the number of fetal resorptions in aged dams, suggesting that TUDCA can have a beneficial effect on pregnancy outcomes with advanced maternal age. Moreover, there was no (negative) impact of TUDCA on pregnancy outcomes in the young dams. Of note, TUDCA improved fetal body weight in aged dams without changes in the placental weight. In general, the major determinant of fetal growth is the ability of the placenta to supply nutrients and oxygen via simple diffusion and/or using various transporter-mediated systems, such as glucose and amino acid transport system [65,66,67,68]. Indeed, there are numerous studies demonstrating placental insufficiency (inability of the placenta to supply sufficient nutrients and oxygen to the fetus) as the primary cause of fetal growth restriction [66,69,70]. Studies showed that ER stress and ROS can directly impact mammalian targets of rapamycin and O-GlcNAc transferase pathways, two key nutrient-sensing proteins involved in amino-acid and glucose transport, causing fetal growth restriction [27,71,72,73]. By reducing placental ER and oxidative stress, TUDCA could thus indirectly control these key nutrient sensors in aged dams to improve fetal weight, however, this remains to be studied in future experiments.

The higher number of fetal resorptions in aged control dams could be attributed to various reasons; impaired decidualization or reduced uterine prostaglandins synthesis that may affect trophoblast cells invasion and eventually reduce the ability of the blastocyst implantation [74,75]. Further, in vitro studies showed that ER stress negatively affects blastocyst formation, decreases blastocyst development, and reduces oocyte maturation and embryo development [25,26,76]. Lin et al. demonstrated that the TUDCA supplementation of the embryo culture medium improved the rate of implantation/number of livebirth rates of transferred mouse embryos in surrogate mice [40]. Thus, increased ER stress could be linked to the higher number of resorptions observed in aged dams, and the reduced resorption rate in aged dams after TUDCA treatment suggests that reducing ER stress may (partially) prevent fetal resorptions.

One of the most important physiological changes for normal pregnancy outcomes is the remodeling of the uterine arteries to supply well-oxygenated blood to the developing fetus [77,78,79,80]. Previously, using our advanced maternal age rat model, we demonstrated changes in uterine artery vascular function in advanced maternal age compared to young dams [13]. In the current study, TUDCA tended to improve endothelium-dependent vasodilation responses in aged dams (without effect in young dams), suggesting increased uterine artery ER stress may have impaired uterine artery vascular function. Further, TUDCA may have improved the uterine artery function by reducing the ER stress, similar to what was observed in the mesenteric arteries, but due to the limited tissue amount of the uterine arteries, we could not measure the levels of ER stress markers. ER stress has been shown to impair uterine artery function. For instance, Hu et al. showed that ER stress suppresses Ca^2+^ sparks/STOCs and increases myogenic tone in uterine arteries in an animal model of pregnant sheep acclimatized to high altitude, which was reversed using TUDCA/PERK inhibitor (GSK2606414) [81]. In addition, no changes in vascular responsiveness to the NO-donor SNP (endothelium-independent response) were found between the groups, suggesting the effect of TUDCA treatment was endothelium-dependent. However, there were no differences in NO contribution which suggests that the improved uterine artery relaxation in aged dams after TUDCA treatment is not NO-dependent and may be due to adaptations in other endothelial vasodilatory pathways (an area that remains to be investigated). Overall, our data indicates that TUDCA improved uterine artery vascular function and adverse pregnancy outcomes in complicated pregnancies.

## 5. Conclusions

Advanced maternal age is associated with an increased risk of pregnancy complications such as fetal growth restriction, preeclampsia, preterm birth, and stillbirth. Our study demonstrated the presence of ER stress in mesenteric arteries from rats of advanced maternal age, and that inhibition of ER stress by TUDCA reduced expressions of ER stress proteins in the mesenteric vasculature. TUDCA treatment also reduced blood pressure, improved fetal body weight, and uterine artery function in aged dams, signifying its beneficial role in advanced maternal age pregnancies. Clinically, maternal aging is frequently associated with co-morbidities, such as hypertension, diabetes, obesity, or cardiovascular disease-associated endothelial dysfunction, therefore, designing future studies that include a second hit (such as high salt or high-fat diet or chemically induced ER stress) are warranted to confirm the potential benefit of TUDCA in these complex pregnancies. In summary, our studies are the first to illustrate the role of ER stress in pregnancies at an advanced maternal age, and for TUDCA as a potential therapeutic that may benefit pregnancy outcomes in this high-risk population.

## Data Availability

Data is contained within the article.
